# Studies on solubility measurement of codeine phosphate (pain reliever drug) in supercritical carbon dioxide and modeling

**DOI:** 10.1038/s41598-023-48234-x

**Published:** 2023-11-29

**Authors:** Gholamhossein Sodeifian, Chandrasekhar Garlapati, Maryam Arbab Nooshabadi, Fariba Razmimanesh, Armin Roshanghias

**Affiliations:** 1 Department of Chemical Engineering, Faculty of Engineering, University of Kashan, Kashan, 87317-53153 Iran; 2 Laboratory of Supercriritcal Fluids and Nanotechnology, University of Kashan, Kashan, 87317-53153 Iran; 3 Modeling and Simulation Centre, Faculty of Engineering, University of Kashan, Kashan, 87317-53153 Iran; 4 Department of Chemical Engineering, Puducherry Technological University, Puducherry, 605014 India; 5 Bolvar Ghotbe Ravandi, Kashan Branch, Islamic Azad University, Ostaadan Street, Kashan, 87159-98151 Iran

**Keywords:** Chemical engineering, Chemical engineering

## Abstract

In this study, the solubilities of *codeine phosphate*, a widely used pain reliever, in supercritical carbon dioxide (SC-CO_2_) were measured under various pressures and temperature conditions. The lowest determined mole fraction of *codeine phosphate* in SC-CO_2_ was 1.297 × 10^−5^ at 308 K and 12 MPa, while the highest was 6.502 × 10^−5^ at 338 K and 27 MPa. These measured solubilities were then modeled using the equation of state model, specifically the *Peng-Robinson model*. A selection of density models, including the *Chrastil model*, *Mendez-Santiago and Teja model*, *Bartle et al. model*, *Sodeifian et al. model*, and *Reddy-Garlapati model*, were also employed. Additionally, three forms of solid–liquid equilibrium models, commonly called expanded liquid models (*ELMs*), were used. The average solvation enthalpy associated with the solubility of *codeine phosphate* in SC-CO_2_ was calculated to be − 16.97 kJ/mol. The three forms of the *ELMs* provided a satisfactory correlation to the solubility data, with the corresponding average absolute relative deviation percent (AARD%) under 12.63%. The most accurate *ELM model* recorded AARD% and AICc values of 8.89% and − 589.79, respectively.

## Introduction

The importance of supercritical fluids (*SCFs*) as solvents in various processes has been recognized for decades^1,2^. Significant applications of *SCFs* encompass particle sizing, extraction, reactions, and separations^3^. *SCFs* serve as solvents in all of these applications^3–6^. However, it is essential to note that while theoretically, all substances can attain a supercritical state, some necessitate exceedingly high pressures and temperatures to achieve this state, rendering it impractical and resource-intensive^7–10^. Carbon dioxide is a well-known substance that readily reaches its supercritical state with minimal effort^11–13^. Consequently, *CO*_*2*_ as an *SCF* is extensively documented in the literature^14–16^.

The sizing of drug particles, whether at the micro or nano level, primarily depends on their solubility^17–20^. The desired drug particle size can be achieved by rapidly expanding supercritical solutions (RESS) or anti-solvent processes^21–23^. The size of a drug particle can play a crucial role in treating various illnesses, as it significantly influences bioavailability^24–28^. Therefore, determining solubility is a fundamental step in micronization/nanonization. While recent literature reports the solubility of codeine phosphate in conventional solvents, information regarding its solubility in *SCFs* is notably absent^29–31^. Hence, this study focuses on measuring the solubility of codeine phosphate in supercritical carbon dioxide (*SC-*CO_2_) under various conditions. A modeling task is also undertaken to facilitate the application of the acquired data.

Several methodologies are available in the literature for modeling solubility data; however, only three are considered user-friendly^32–34^. The first method involves the use of the Equation of State (*EoS*), which requires critical properties of both the solute (the drug) and the solvent (*SC-CO*_*2*_). The second method relies on semi-empirical models, often referred to as density-based models, which necessitate data on the density of the solvent, as well as temperature and pressure data. The final method is the solid–liquid equilibrium model, also known as the expanded liquid model (*ELM*), which requires information about the solute's enthalpy of melting and the solute's melting temperature^35–38^.To obtain the required properties such as critical temperature, critical pressure, acentric factor, molar volume, and sublimation pressures, standard group contribution techniques are employed^39–41^.However, there are instances where the application of group contribution methods becomes challenging due to the absence of functional group contributions, such as phosphate and sulfates. Applying EoS modeling and the solid–liquid equilibrium model can prove challenging^42–45^.Codeine phosphate, an analgesic drug, exemplifies such a compound where critical properties ($T_{c}$ and $P_{c}$), molar volume($v_{2}$)and sublimation pressures are unavailable, and existing group contribution techniques cannot be applied due to the presence of phosphate in its structure. However, experimental data for the melting temperature (155 °C) and the heat of fusion (18.86 cal/g or 78.91 J/g or 31,358.83 J/mol) of codeine phosphate are readily accessible^46–48^. The magnitude of codeine phosphate's solubility in *SC-CO*_*2*_ determines the technique employed for drug micronization/nanonization using *SC-CO*_*2*_.

The present work unfolds in two distinct phases. In the first phase, the solubilities of codeine phosphate in *SC-CO*_*2*_ are measured under various conditions. The second phase evaluates the collected data using EoS, density, and ELM models.

## Experiment section

### Materials

*Codeine phosphate* was provided by *Parsian Pharmaceutical Co.* (Tehran, Iran) with a *CAS number* of 52-28-8 and a mass purity exceeding 99%. *CO*_*2*_ (carbon dioxide) with a *CAS number* of 124-38-9 and a mass purity exceeding 99.9% was supplied by *Fadak Company*, Kashan, Iran. Table 1 provides information about the chemicals used in this study.

**Table 1:** Molecular structure and physiochemical properties of used materials.

Compound	Formula	Structure	M_W_ (g/mol)	λ_max_ (nm)	CAS number	Minimum purity Mass fraction
Codeine phosphate	C_18_H_21_NO_3_. H_3_PO_4_		397.4	281	52-28-8	99%
Carbon dioxide	CO_2_		44.01		124-38-9	0.9999

### Equipment details

**Figure 1:**
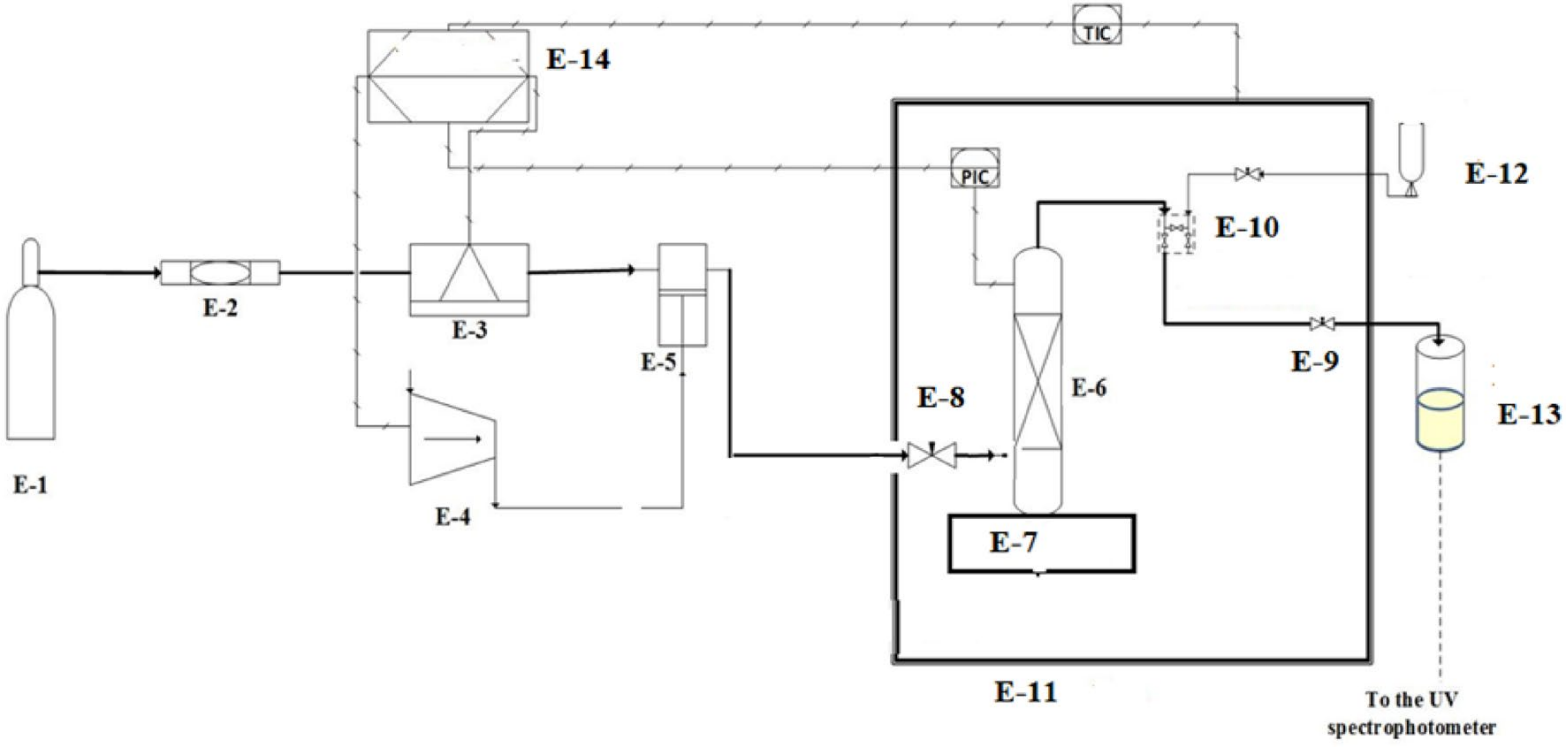
Experimental setup for solubility measurement, E1- CO_2_ cylinder; E2- Filter; E3- Refrigerator unit; E4- Air compressor; E5- High pressure pump; E6- Equilibrium cell; E7- Magnetic stirrer; E8- Needle valve; E9- Back-pressure valve; E10- Six-port, two position valve; E11- Oven; E12- Syringe; E13- Collection vial; and E14- Control panel.

Static equipment was employed for solubility measurements, as depicted in Fig. 1. Comprehensive equipment details can be found in our previous studies^49–51^. This section offers a concise explanation of the experimental setup and methodology. Thermodynamically, the measurement method falls under the category of *isobaric-isothermal* methods^52^. Throughout the experiments, temperatures and pressures were rigorously controlled at the desired experimental conditions with a precision of ± 0.1 K for temperature and ± 0.1 MPa for pressure, respectively. Solubility measurements were conducted in triplicate for each data point. In each measurement, a known quantity of *codeine phosphate drug* (1 g) was utilized, and after reaching equilibrium, the saturated sample was collected through a *2-position 6-way port valve* into a vial preloaded with *demineralized water* (*DM water*). After discharging 600 µL of *saturated SC-CO*_*2*_ the port valve was rinsed with 1 ml of *DM water*, resulting in a total saturation solution volume of 5 ml.

**Figure 2:**
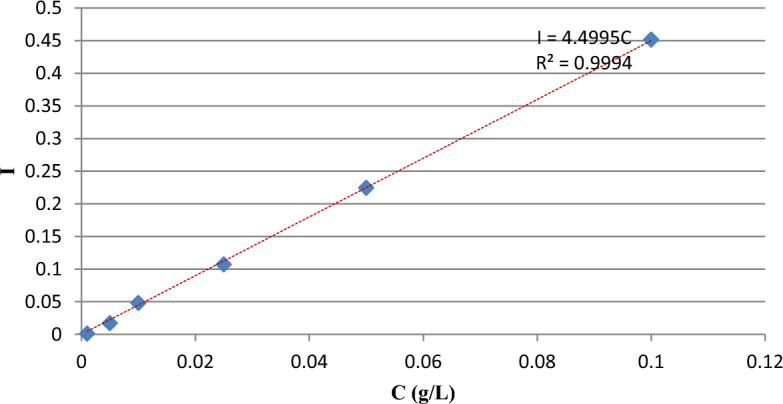
The calibration curve of drug in DM water.

The drug solubility values were measured by absorbance assays at ${\lambda }_{\mathrm{max}}$ (281 nm) on a UNICO-4802 UV–Vis spectrophotometer with 1-cm pass length quartz cells and the solubility was calculated from the concentration of solute by using the calibration curve (with regression coefficient 0.999) and the UV-absorbance, Fig. 2.

For solubility calculations, the following equations were employed:

1 $$y_{2} = \frac{{n_{drug} }}{{n_{drug} + n_{{CO_{2} }} }}$$

where ${n}_{\text{drug}}$ and ${n}_{{\text{CO}}_{2}}$ represent the moles of codeine phosphate and CO_2_, respectively.

Moreover, these quantities are defined as follows:

2 $${n}_{\text{drug}}=\frac{{C}_{s}\cdot {V}_{s}}{{M}_{s}}$$

3 $${n}_{{\text{CO}}_{2}}=\frac{{V}_{1}\cdot \rho }{{M}_{{\text{CO}}_{2}}}$$

In the above relations, ${C}_{\text{s}}$ is defined as the drug concentration in saturated sample vial in g/L. Also, the volume of the sampling loop and vial collection are expressed as V_1_(L) = 600 $\times$ 10^–6^ m^3^ and V_s_(L) = 5 $\times$ 10^–3^ m^3^, respectively. The $M_{s}$ and $M_{{CO_{2} }}$ are the molecular weights of the codeine phosphate drug (component 2) and CO_2_, respectively.

Solubility can be also expressed as:

4 $$S = \frac{{C_{S} V_{s} }}{{V_{1} }}$$

where, one can find the relation between S and $y_{2}$ as follows:

5 $$S = \frac{{\rho M_{s} }}{{M_{{CO_{2} }} }}\frac{{y_{2} }}{{1 - y_{2} }}$$

*Codeine phosphate*’s solubility was determined using a *UV–visible spectrophotometer* (*Model UNICO-4802*, double beam, with multipurpose software, USA), with *DM water* as the solvent.

## Modeling

The solubility data obtained in this study were correlated with one equation of state *(EoS)*, five density-based models, and three ELM models. we considered the *Peng-Robinson (PR) EoS*. In the case of density-based modeling, several well-known models, namely *Chrastil*, *Mendez-Santiago, Teja (MT)*, *Bartle *et al*.*, *Sodeifian *et al*.*, and *Reddy-Garlapati* were employed. Three forms of *ELMs* with different parameters were used for data fitting. Detailed information about all the models considered in this work is discussed in the following sections.

### EoS modeling

This model is an extension to the model framework suggested by Schmitt^53^ and Reid and Estévez et al.^54^. PR EoS was used for the modeling. Solubility of codeine phosphate (solute, component 2) in SC-CO_2_ (solvent, component 1) is expressed as^55^

6 $${\text{y}}_{{2}} = \frac{{P_{2}^{S} \hat{\phi }_{2}^{S} }}{{P\hat{\phi }_{2}^{{SC - CO_{2} }} }}\exp \left[ {\frac{{\left( {P - P_{2i}^{S} } \right)v_{S} }}{RT}} \right] \,$$

where $P_{2}^{s}$, $\hat{\phi }_{2}^{{SC - CO_{2} }}$, $\hat{\phi }_{2}^{S}$ $P$, $v_{2}$, $T$ and *R,* are sublimation pressure, solid solute fugacity coefficient, saturation fugacity coefficient, system pressure, drug molar volume, system temperature and universal gas constant, respectively. The required equation for the solid solute fugacity coefficient in the SC-CO_2_ ($\hat{\phi }_{2}^{{SC - CO_{2} }}$) is calculated using PR EoS. It is obtained from the following thermodynamic equation.

7 $$\ln \left( {\hat{\varphi }_{2}^{{SC - CO_{2} }} } \right) = \frac{1}{RT}\int\limits_{v}^{\infty } {\left[ {\left( {\frac{\partial P}{{\partial N_{i} }}} \right)_{{T,V,N_{1} }} - \frac{RT}{v}} \right]} dv - \ln Z$$

Equation (8) represents the fugacity coefficients expression for PR EoS.

8 $$\ln \left( {\hat{\varphi }_{2}^{{SC - CO_{2} }} } \right) = {{b_{2} } \mathord{\left/ {\vphantom {{b_{2} } b}} \right. \kern-0pt} b}\left( {Z - 1} \right) - \ln \left( {P\left( {V - b} \right)/RT} \right) - {a \mathord{\left/ {\vphantom {a {(2\sqrt 2 }}} \right. \kern-0pt} {(2\sqrt 2 }}RTb)\left\{ {[2(a_{12} y_{1} + a_{2} y_{2} )/a] - {{b_{2} } \mathord{\left/ {\vphantom {{b_{2} } b}} \right. \kern-0pt} b}} \right\}\ln \left[ {\frac{V + 2.414b}{{V - 0.414b}}} \right]$$

For modeling tasks, critical temperature, critical pressure, centric factor, molar volume, and sublimation pressures of the codeine phosphate are required. Unfortunately, they are unavailable for this typical drug. Therefore, to overcome this drawback, the following assumptions are applied.

#### Assumption 1

Solute in the solvent is very dilute. Thus, the required $\hat{\phi }_{2}^{{SC - CO_{2} }}$ is obtained by applying William J. Schmitt and Robert C. Reid assumptions to Eq. (8) (i.e., for dilute system $z \to z_{1}$, $a \to a_{1}$ and $b \to b_{1}$). Thus, $\hat{\phi }_{2}^{{SC - CO_{2} }}$ PR EoS (Eq. 8) is reduced to Eq. (9). In which solute parameters are adjustable (i.e., $a_{2}$ and $b_{2}$)^53^.

9 $$\ln \left( {\hat{\varphi }_{2}^{{SC - CO_{2} }} } \right) \approx {{b_{2} } \mathord{\left/ {\vphantom {{b_{2} } b}} \right. \kern-0pt} b}_{1} \left( {Z_{1} - 1} \right) - \ln \left( {P\left( {V_{1} - b_{1} } \right)/RT} \right) - {{a_{1} } \mathord{\left/ {\vphantom {{a_{1} } {(2\sqrt 2 }}} \right. \kern-0pt} {(2\sqrt 2 }}RTb_{1} )\left\{ {[2(a_{2} )/a_{1} ] - {{b_{2} } \mathord{\left/ {\vphantom {{b_{2} } {b_{1} }}} \right. \kern-0pt} {b_{1} }}} \right\}\ln \left[ {\frac{{V_{1} + 2.414b_{1} }}{{V_{1} - 0.414b_{1} }}} \right]$$

#### Assumption 2

The molar volume of solute ($v_{2}$) is a function of SC-CO_2_ (solvent) density ($\rho_{1}$)^56^ and in this work the following expression is used

10 $$v_{2} = K_{1} + K_{2} \rho_{1} + K_{3} \rho_{1}^{2}$$

where $$K_{1} ,\,$$$$K_{2} ,\,$$$$K_{3}$$ have units are m^3^/mol, m^6^/mol kg, m^9^/mol kg^2^, respectively.

#### Assumption 3

The sublimation pressure of the solute is expressed as a function of temperature, and it is expressed as Eq. (11)^55^

11 $$R\ln \left( {P_{A}^{sub} } \right) = \beta + \frac{\gamma }{T} + \Delta_{sub} \delta \ln \left( {\frac{T}{298.15}} \right)$$

where $\beta$, $\gamma$ and $\Delta_{sub} \delta$ are sublimation pressure expression coefficients. They are substituting Eqs. (9)–(11), in Eq. (6), results in the solubility model based on PR EoS in terms of pressure, temperature, density, and some adjustable parameters. The adjustable parameters are $a_{2}$, $b_{2}$, $\beta$, $\gamma$,$\Delta_{sub} \delta$,$K_{1}$,$K_{2}$ and $K_{3}$. These parameters are treated as temperature-independent in the temperature range considered in the present work. The adjustable parameters are obtained by regression with experimental data.

For the data regression, the objective function, Eq. (12), is used^57^

12 $$OF = \sum\limits_{i = 1}^{N} {\frac{{\left| {y_{2i}^{\exp } - y_{2i}^{calc} } \right|}}{{y_{2i}^{\exp } }}}$$

where $y_{2i}^{\exp }$ is the experimental mole fraction of solute, and $y_{2i}^{calc}$ is the model predicted mole fraction of solute.

### Density-based modeling

#### Chrastil model^58–60^

Solute concentration and solvent density are related as follows:

13 $$c_{m} = \left( {\rho_{m1} } \right)^{\kappa } \exp \left( {A_{1} + {\raise0.7ex\hbox{${B_{1} }$} \!\mathord{\left/ {\vphantom {{B_{1} } {{T \mathord{\left/ {\vphantom {T K}} \right. \kern-0pt} K}}}}\right.\kern-0pt} \!\lower0.7ex\hbox{${{T \mathord{\left/ {\vphantom {T K}} \right. \kern-0pt} K}}$}}} \right)$$

where $c_{m}$ is the mass concentration of solute,$\rho_{m1}$ is the mass concentration of solvent, and $\kappa$,$A_{1}$ and $B_{1}$ are model constants.

Equation (1) can be rearranged to mole fraction as follows:

14 $$\frac{{c_{m} }}{{\rho_{m1} }}\frac{{M_{ScF} }}{{M{}_{Solute}}} = \frac{{M_{ScF} }}{{M{}_{Solute}}}\left( {\rho_{1} } \right)^{\kappa - 1} \exp \left( {A_{1} + {\raise0.7ex\hbox{${B_{1} }$} \!\mathord{\left/ {\vphantom {{B_{1} } {{T \mathord{\left/ {\vphantom {T K}} \right. \kern-0pt} K}}}}\right.\kern-0pt} \!\lower0.7ex\hbox{${{T \mathord{\left/ {\vphantom {T K}} \right. \kern-0pt} K}}$}}} \right)$$

where $M_{ScF}$, $M{}_{Solute}$ and $c_{m} /M_{solute}$ are molar mass of SCF, molar mass of solute, and molar concentration of solute ($c$), respectively. Also,$\rho_{m1} /M_{ScF}$ and $\kappa$ are molar concentration of solvent($\rho_{1}$), and association number, respectively. Furthermore, $A_{1}$ and $B_{1}$ are model constants.

15 $$mole\;ratio = \frac{c}{{\rho_{1} }} = \frac{{M_{ScF} }}{{M{}_{Solute}}}\left( {\rho_{1} } \right)^{\kappa - 1} \exp \left( {A_{1} + {\raise0.7ex\hbox{${B_{1} }$} \!\mathord{\left/ {\vphantom {{B_{1} } {{T \mathord{\left/ {\vphantom {T K}} \right. \kern-0pt} K}}}}\right.\kern-0pt} \!\lower0.7ex\hbox{${{T \mathord{\left/ {\vphantom {T K}} \right. \kern-0pt} K}}$}}} \right)$$

Mole fraction ($y_{2}$) and mole ratios are related as follows:

16 $$\frac{c}{{\rho_{1} }} = \frac{{y_{2} }}{{1 - y_{2} }}$$

17 $$y_{2} = {{mole\;ratio} \mathord{\left/ {\vphantom {{mole\;ratio} {\left[ {1 + mole\;ratio} \right]}}} \right. \kern-0pt} {\left[ {1 + mole\;ratio} \right]}}$$

18 $$y_{2} = {{\frac{{M_{ScF} }}{{M{}_{Solute}}}\left( {\rho_{1} } \right)^{\kappa - 1} \exp \left( {A_{1} + {\raise0.7ex\hbox{${B_{1} }$} \!\mathord{\left/ {\vphantom {{B_{1} } {{T \mathord{\left/ {\vphantom {T K}} \right. \kern-0pt} K}}}}\right.\kern-0pt} \!\lower0.7ex\hbox{${{T \mathord{\left/ {\vphantom {T K}} \right. \kern-0pt} K}}$}}} \right)} \mathord{\left/ {\vphantom {{\frac{{M_{ScF} }}{{M{}_{Solute}}}\left( {\rho_{1} } \right)^{\kappa - 1} \exp \left( {A_{1} + {\raise0.7ex\hbox{${B_{1} }$} \!\mathord{\left/ {\vphantom {{B_{1} } {{T \mathord{\left/ {\vphantom {T K}} \right. \kern-0pt} K}}}}\right.\kern-0pt} \!\lower0.7ex\hbox{${{T \mathord{\left/ {\vphantom {T K}} \right. \kern-0pt} K}}$}}} \right)} {\left[ {1 + \frac{{M_{ScF} }}{{M{}_{Solute}}}\left( {\rho_{1} } \right)^{\kappa - 1} \exp \left( {A_{1} + {\raise0.7ex\hbox{${B_{1} }$} \!\mathord{\left/ {\vphantom {{B_{1} } {{T \mathord{\left/ {\vphantom {T K}} \right. \kern-0pt} K}}}}\right.\kern-0pt} \!\lower0.7ex\hbox{${{T \mathord{\left/ {\vphantom {T K}} \right. \kern-0pt} K}}$}}} \right)} \right]}}} \right. \kern-0pt} {\left[ {1 + \frac{{M_{ScF} }}{{M{}_{Solute}}}\left( {\rho_{1} } \right)^{\kappa - 1} \exp \left( {A_{1} + {\raise0.7ex\hbox{${B_{1} }$} \!\mathord{\left/ {\vphantom {{B_{1} } {{T \mathord{\left/ {\vphantom {T K}} \right. \kern-0pt} K}}}}\right.\kern-0pt} \!\lower0.7ex\hbox{${{T \mathord{\left/ {\vphantom {T K}} \right. \kern-0pt} K}}$}}} \right)} \right]}}$$

where $\kappa$,$A_{1}$ and $B_{1}$ are the model constants and their units are dimensionless, dimensionless and K, respectively.

#### Méndez-Santiago and Teja (MT) model^61^

This model can generally be used for checking thermodynamic consistency. It is stated as Eq. (19) and when $T\ln \left( {y_{2} P} \right) - C_{2} T$ vs. $\rho_{1}$ is established, all data points fall around a single straight line

19 $$T\ln \left( {y_{2} P} \right) = A_{2} + B_{2} \cdot \rho_{1} + C_{2} T$$

where $A_{2}$, $B_{2}$ and $C_{2}$ are the model constants and their units are K, K m^3^/kg and dimensionless, respectively

#### Bartle et al. model^62^

According to the model, the solubility is expressed as Eq. (20)

20 $$\ln \left( {\frac{{y_{2} \cdot P}}{{P_{ref} }}} \right) = A_{3} + \frac{{B_{3} }}{T} + C_{3} \left( {\rho_{1} - \rho_{ref} } \right)$$

where the pressure ($P_{ref}$) and density for reference states ($\rho_{ref}$) are considered 0.1 MPa, and 700 kg m^−3^. Also, $A_{3}$, $B_{3}$ and $C_{3}$ are the model constants and their units are dimensionless, K and m^3^/kg, respectively. From the constant $B_{3}$, sublimation enthalpy can be obtained (i.e.,$\Delta_{sub} H = - B_{3} R$ J/mol).

#### Sodeifian et al. model^63^

According to this model, the solubility is represented by Eq. (21)

21 $$y_{2} = A_{4} + B_{4} \frac{{P^{2} }}{T} + C_{4} \ln \left( {\rho_{1} T} \right) + D_{4} \rho_{1} \ln \left( {\rho_{1} } \right) + E_{4} P\ln \left( T \right) + F_{4} \frac{{\ln \left( {\rho_{1} } \right)}}{T}$$

where $A_{4}$, $B_{4}$, $C_{4}$, $D_{4}$, $E_{4}$ and $F_{4}$ are the model constants and their units are dimensionless, K/MPa^2^, dimensionless, m^3^/Kg, 1/MPa and K, respectively.

#### Reddy-Garlapati model^64^

According to the model, the solubility is expressed as Eq. (22)

22 $$y_{2} = \left( {A_{5} + B_{5} P_{r} + C_{5} P_{r}^{2} } \right)T_{r} + (D_{5} + E_{5} P_{r} + F_{5} P_{r}^{2} )$$

where $A_{5}$ $B_{5}$, $C_{5}$, $D_{5}$, $E_{5}$ and $F_{5}$ are the model constants and all are dimensionless quantities;$P_{r}$ is reduced pressure and $T_{r}$ is reduced temperature.

### Expanded Liquid Models (ELMs)

This section deals with models under the solid–liquid equilibrium model (also known as ELMs). It relies on the solution theory, where SC-CO_2_ was considered an expanded liquid with infinite dissolved codeine phosphate. The essential solubility expression is given by^65–68^

23 $$y_{2} = \frac{1}{{\gamma_{2}^{\infty } }}\frac{{f_{2}^{S} }}{{f_{2}^{L} }}$$

where $\gamma_{2}^{\infty }$ is the activity coefficient of solute at infinite dilution,$f_{2}^{S}$, $f_{2}^{L}$ are fugacity of codeine phosphate compound in the solid phase and expanded liquid phase, respectively. The basic equation for the fugacity ratio is represented by

24 $$\ln \left( {\frac{{f_{2}^{S} }}{{f_{L}^{L} }}} \right) = \frac{{\Delta H_{2}^{m} }}{RT}\left( {\frac{T}{{T_{m} }} - 1} \right) - \int\limits_{{T_{m} }}^{T} {\frac{1}{{RT^{2} }}} \left[ {\int\limits_{{T_{m} }}^{T} {\left[ {\Delta C_{p} } \right]dT} } \right]dT$$

where $\Delta C_{p}$ implies the difference between the heat capacity of solid and expanded liquid states. When Eqs. (23 and 24) are combined, the solubility expression for ELM is obtained as Eq. (25)

25 $$y_{2} = \frac{1}{{\gamma_{2}^{\infty } }}\exp \left[ {\frac{{\Delta H_{2}^{m} }}{RT}\left( {\frac{T}{{T_{m} }} - 1} \right) - \int\limits_{{T_{m} }}^{T} {\frac{1}{{RT^{2} }}} \left[ {\int\limits_{{T_{m} }}^{T} {\left[ {\Delta C_{p} } \right]dT} } \right]dT} \right]$$

The solubility expression may be estimated with and without $\Delta C_{p}$ term. In the following section, three cases are presented. For all three cases, a unique expression for $\gamma_{2}^{\infty }$ used^23,69^ was $\exp \left( {l_{1} + l_{2} (p/(RT)} \right) + l_{3} (p/(RT))^{2} )$.

*Case 1*. $$\Delta C_{p} = 0$$.

The solubility expression for this case is written as

26 $$y_{2} = \exp \left[ {\frac{{\Delta H_{2}^{m} }}{{RT}}\left( {\frac{T}{{T_{m} }} - 1} \right)} \right]/\exp \left( {l_{1} + l_{2} \left( {\frac{p}{{RT}}} \right) + l_{3} \left( {\frac{p}{{RT}}} \right)^{2} } \right)$$

Thus Eq. (26) has three maximum parameters (*l*_1_, *l*_2_ and *l*_3_).

*Case 2*. $\Delta C_{p} = {\text{contant}}$. Consider the constant $\Delta C_{p}$ is D^23^.

The solubility expression for this case iswritten as

27 $$y_{2} = \exp [\frac{{\Delta H_{2}^{m} }}{{RT}}\left( {\frac{T}{{T_{m} }} - 1} \right) - {\raise0.7ex\hbox{$D$} \!\mathord{\left/ {\vphantom {D R}}\right.\kern-\nulldelimiterspace} \!\lower0.7ex\hbox{$R$}}\left[ {\ln \left( {\frac{T}{{T_{m} }}} \right) - T_{m} \left( {\frac{1}{{T_{m} }} - \frac{1}{T}} \right)} \right]/\exp \left( {l_{1} + l_{2} \left( {\frac{p}{{RT}}} \right) + l_{3} \left( {\frac{p}{{RT}}} \right)^{2} } \right)$$

Thus Eq. (27) has four maximum of parameters ($D$, *l*_1_, *l*_2_ and *l*_3_) and respective units are J/mole K, dimensionless, J/mole MPa and J^2^/mole^2^ MPa^2^, respectively.

*Case 3*. $$\Delta C_{p} = f(T)$$.

Generally,$C_{p}$ it is a third-order polynomial equation in temperature equation; however, a recent study on solubility modeling shows that a good fit is achieved with the second-order polynomial. Thus, it is assumed that the $\Delta C_{p}$ quadratic function in temperature as Eq. (28)^70^

28 $$\Delta C_{p} = \beta_{1} + \beta_{2} T + \beta_{3} T^{2}$$

Integral evaluation of Eq. (25) by substituting Eq. (28) results in Eq. (29)

29 $$\begin{aligned} \ln \left( {\frac{{f_{2}^{S} }}{{f_{L}^{L} }}} \right) &= \frac{{\Delta H_{2}^{m} }}{{RT}}\left( {\frac{T}{{T_{m} }} - 1} \right) - \frac{{\beta _{1} }}{R}\left[ {\ln \left( {\frac{T}{{T_{m} }}} \right) - T_{m} \left( {\frac{1}{{T_{m} }} - \frac{1}{T}} \right)} \right] - \frac{{\beta _{2} }}{{2R}}\left[ {\left( {T - T_{m} } \right) - T_{m}^{2} \left( {\frac{1}{{T_{m} }} - \frac{1}{T}} \right)} \right] \hfill \\ &\quad - \frac{{\beta _{3} }}{{3R}}\left[ {\left( {\frac{{T^{2} }}{2} - \frac{{T_{m}^{2} }}{2}} \right) - T_{m}^{3} \left( {\frac{1}{{T_{m} }} - \frac{1}{T}} \right)} \right] \hfill \\ \end{aligned}$$

Thus, the solubility expression for this case is written as

30 $$\begin{gathered} y_{2} = \exp [\frac{{\Delta H_{2}^{m} }}{RT}\left( {\frac{T}{{T_{m} }} - 1} \right) - \frac{{\beta_{1} }}{R}\left[ {\ln \left( {\frac{T}{{T_{m} }}} \right) - T_{m} \left( {\frac{1}{{T_{m} }} - \frac{1}{T}} \right)} \right] - \frac{{\beta_{2} }}{2R}\left[ {\left( {T - T_{m} } \right) - T_{m}^{2} \left( {\frac{1}{{T_{m} }} - \frac{1}{T}} \right)} \right] \hfill \\ \;\;\;\;\;\;\;\;\;\;\;\; - \frac{{\beta_{3} }}{3R}\left[ {\left( {\frac{{T^{2} }}{2} - \frac{{T_{m}^{2} }}{2}} \right) - T_{m}^{3} \left( {\frac{1}{{T_{m} }} - \frac{1}{T}} \right)} \right]]/\exp \left( {l_{1} + l_{2} \left( \frac{p}{RT} \right) + l_{3} \left( \frac{p}{RT} \right)^{2} } \right)] \hfill \\ \end{gathered}$$

In Eq. (30), six parameters are there and they are $\beta_{1}$,$\beta_{2}$,$\beta_{3}$, *l*_1_, *l*_2_ and *l*_3_ and their units are J/K kg, J/K^2^kg, J/K^3^kg, dimensionless, J/mole MPa and J^2^/mole^2^ MPa^2^, respectively. These parameters are optimally fitted to experimental solubility data by minimizing the error with the help of the objective function defined in Eq. (12). It is also important to note that all three expressions for solubility are explicit functions of composition.

## Results and discussion

The present study reports the measured solubilities of *codeine phosphate* (*C*_*18*_*H*_*21*_*NO*_*3*_) in supercritical carbon dioxide (*SC-CO*_*2*_) at temperatures of 308, 318, 328, and 338 K, spanning a pressure range of 12–27 MPa. Three types of models mentioned in the previous section were used in data correlation. The correlation task was carried out in *MATLAB 2019®* using the inbuilt *fminsearch* algorithm. The optimization algorithm minimized the error and was used for parameter estimation for all the models mentioned in the previous section. The measured data are shown in Table 2. The solvent density was obtained from the *NIST* database^71^. Considering the order of magnitude of *codeine phosphate* solubility in *SC-CO*_*2*_, supercritical anti-solvent methods can be regarded an appropriate choice for producing fine particles of this drug.

**Table 2:** Solubility of crystalline codeine phosphatein SC-CO_2_ at various temperatures and pressures.

Temperature (K)^a^	Pressure (MPa)^a^	Density of SC-CO_2_ (kg/m^3^)^1^	y_2_ × 10^5^ (Mole fraction)	Experimental standard deviation, S(ȳ) × (10^5^)	S (Equilibrium solubility) (g/L)	Expanded uncertainty of Mole fraction (10^5^ U)
308	12	769	1.297	0.021	0.090	0.072
	15	817	1.615	0.014	0.119	0.078
	18	849	1.702	0.022	0.131	0.086
	21	875	1.754	0.083	0.138	0.113
	24	896	1.897	0.031	0.153	0.104
	27	914	1.991	0.055	0.164	0.103
318	12	661	1.387	0.038	0.083	0.099
	15	744	2.614	0.091	0.176	0.041
	18	791	2.742	0.021	0.196	0.130
	21	824	3.158	0.077	0.235	0.108
	24	851	3.422	0.093	0.263	0.109
	27	872	3.817	0.040	0.300	0.106
328	12	509	1.891	0.082	0.087	0.115
	15	656	3.594	0.011	0.213	0.124
	18	725	3.949	0.088	0.259	0.150
	21	769	4.284	0.062	0.298	0.127
	24	802	4.521	0.095	0.327	0.176
	27	829	5.441	0.063	0.407	0.171
338	12	388	2.294	0.071	0.080	0.178
	15	557	3.959	0.081	0.199	0.142
	18	652	4.395	0.033	0.259	0.108
	21	710	4.927	0.128	0.316	0.137
	24	751	5.521	0.141	0.375	0.173
	27	783	6.502	0.065	0.459	0.115

The experimental standard deviation was obtained by $$S\left({y}_{k}\right)=\sqrt{\frac{\sum_{j=1}^{n}{\left({y}_{j}-\overline{y }\right)}^{2}}{n-1}}$$. Expanded uncertainty (U) and the relative combined standard uncertainty (u_combined_/y) are defined, respectively, as follows: (U) = k*u_*combined*_ (k = 2) and $${\raise0.7ex\hbox{${u_{{combined}} }$} \!\mathord{\left/ {\vphantom {{u_{{combined}} } y}}\right.\kern-\nulldelimiterspace} \!\lower0.7ex\hbox{$y$}} = \sqrt {\mathop \sum \limits_{{i = 1}}^{N} \left( {P_{i} u\left( {x_{i} } \right)/x_{i} } \right)^{2} }$$. In this research, *u*(*x*_*i*_) was considered as standard uncertainties of temperature, pressure, mole fraction, volumes and absorption. *P*_*i*_, sensitivity coefficients, are equal to the partial derivatives of y equation (Eq. 1) with respect to the *x*_*i*_.

^a^Standard uncertainty u are u(T) =  ± 0.1 K; u(p) =  ± 1 bar.

The value of the coverage factor k = 2 was chosen on the basis of the level of confidence of approximately 95 percent for calculating the expanded uncertainty.

**Figure 3:**
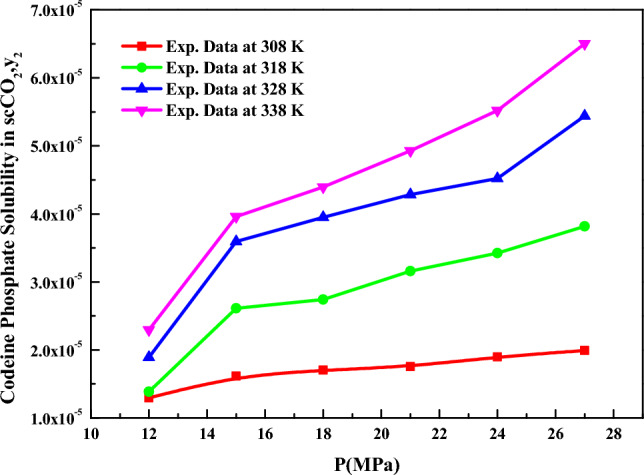
Codeine phosphate solubility in SC-CO_2_, $y_{2}$ versus P (MPa).

The solubility of *codeine phosphate* in *SC-CO*_*2*_ vs. pressure is depicted in Fig. 3. From the solubility plot, it is evident that a cross-over pressure is not observed for *codeine phosphate*. Since conducting experimental investigations at each required condition (pressure and temperature) is tedious, modeling becomes necessary. Therefore, modeling was performed in all three modes. Numerous equations of state (EoS) are available in the literature for modeling solubility data. However, the PR EoS was selected in this work due to its success in modeling the solubilities of solid substances in supercritical fluids (*SCFs*)^53–55,70^. When correlating the data, the *PR EoS* model parameters were treated as temperature-independent over 308–338 K. The objective function indicated in Eq. (12) was utilized for data correlation, and all the adjustable parameters were obtained through regression with experimental data. Table 3 presents the correlation constants of the *PR EoS* model. Sublimation enthalpies at 308, 318, 328, and 338 K were calculated from the vapor pressure expression constants using the following relation:

31 $$\Delta_{sub} H = \left( { - \gamma + \Delta_{sub} \delta \;T} \right)R\;{\text{J}}/{\text{mol}}$$

**Table 3:** Correlation constant of EoS model.

Name of the model	Parameters	AARD%	$R^{2}$	$R_{adj}^{2}$
PREoS	$a_{1}$ = 2.3463 × 10^–4^	8.61	0.918	0.899
	$b_{1}$ = 4.2013 × 10^–4^			
	$\beta$ = 23.310			
	$\gamma$ = − 9127.5			
	$\Delta_{sub} \delta$ = − 5.9988			
	$K_{1}$ = 7.516 × 10^–4^			
	$K_{2}$ = − 7.3793 × 10^–8^			
	$K_{3}$ = − 7.8339 × 10^–11^			
Estimated Sublimation Enthalpies at T (K)	Sublimation Enthalpy (kJ/mol) Estimated using $\Delta_{sub} H = ( - \gamma + \Delta_{sub} \delta \;T)R$	Average Sublimation Enthalpy in kJ/mol		
308	60.524	59.776		
318	60.026			
328	59.527			
338	59.028			

The estimated sublimation enthalpies are presented in Table 3. The correlating ability of the *equation of state (EoS)* method is depicted in Fig. 3.

**Figure 4:**
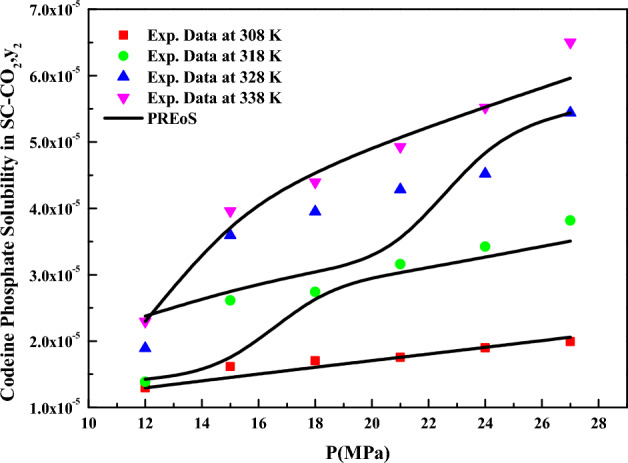
Codeine phosphate solubility in SC-CO_2_, $y_{2}$ versus P (MPa). Symbols are experimental points; lines are PREoS model fit.

When considering density-based models for data correlation, the *Chrastil* model (Eq. 18), treated constants as independent variables, and their values were determined through regression with experimental data. The obtained constants are reported in Table 4. The correlating ability of the *Chrastil* model is illustrated in Fig. 4. Reasonable fit is observed when the data is represented as y_2_ versus $\rho_{1}$, this confirms the applicability of the *Chrastil* model to the solubility data^72,73^. From the parameters of the *Chrastil* model, the total enthalpy for *codeine phosphate* was derived, and its value is reported in Table 5.

**Table 4:** Correlation constant of density-based models.

Name of the model	Parameters	AARD%	$R^{2}$	$R_{adj}^{2}$
Chrastil	$\kappa$ = 2.8403	9.48	0.902	0.897
	$A_{1}$ = − 4.0221			
	$B_{1}$ = − 5284.7			
MT model	$A_{2}$ = − 8817.9	11.8	0.891	0.886
	$B_{2}$ = 17.341			
	$C_{2}$ = 15.802			
Bartle et al.	$A_{3}$ = 17.257	12.3	0.894	0.889
	$B_{3}$ = − 7326			
	$C_{3}$ = 5.2862 × 10^–3^			
Sodeifian et al.	$A_{4}$ = − 0.015589	8.52	0.936	0.933
	$B_{4}$ = − 3.2068 × 10^–5^			
	$C_{4}$ = 0.40046			
	$D_{4}$ = 1.1612 × 10^–3^			
	$E_{4}$ = − 4.7474 × 10^–3^			
	$F_{4}$ = − 1005.1			
Reddy and Garlapati	$A_{5}$ = 1.68 × 10^–4^	9.48	0.954	0.952
	$B_{5}$ = − 1.2939 × 10^–6^			
	$C_{5}$ = 2.2027 × 10^–5^			
	$D_{5}$ = − 2.0904 × 10^–4^			
	$E_{5}$ = 3.8763 × 10^–5^			
	$F_{5}$ = − 2.8153 × 10^–5^

**Table 5:** Thermodynamic parameters of codeine phosphate-SC-CO_2_ system.

Model	Name of property		
	Total enthalpy ΔH_total_ (kJ/mol)	Enthalpy of sublimation ΔH_sub_ (kJ/mol)	Enthalpy of solvation ΔH_sol_ (kJ/mol)
Chrastil model	43.94^a^		− 16.97^d^
Bartle et al. model		60.91^b^ (average value)	− 15.84^e^
PREoS		59.78^c^ (average value)	

^d^Obtained as a result of difference between ΔH_sub_^b^ and ΔH_total_^a^.

^e^Obtained as a result of difference between ΔH_sub_^c^ and ΔH_total_^a^.

**Figure 5:**
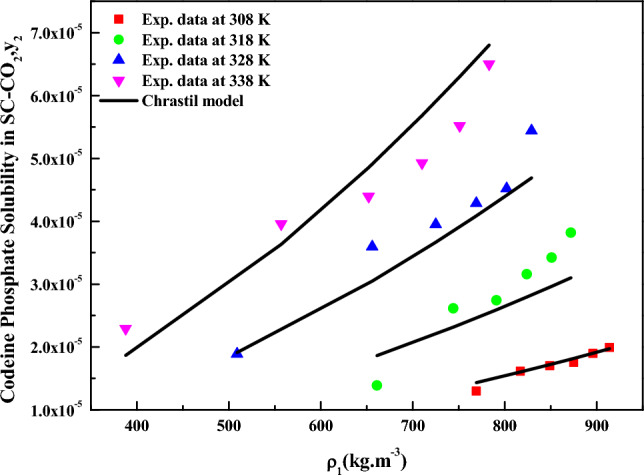
Codeine Phosphate Solubility, y_2_ versus $\rho_{1}$. Symbols are experimental points, and lines are Chrastil model fit.

**Figure 6:**
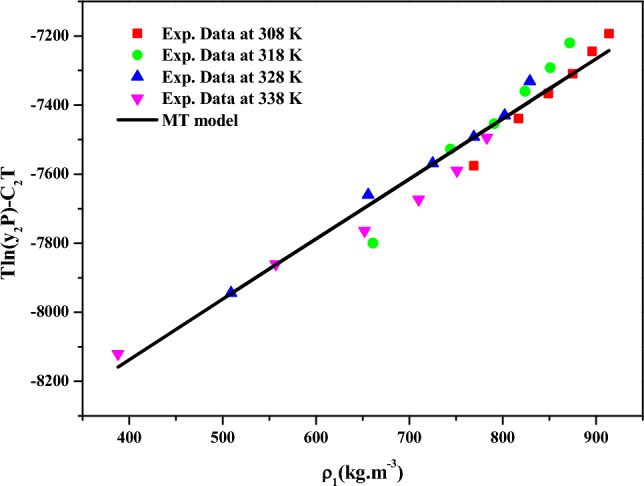
$T\ln (y_{2} P) - C_{2} T$ vs. $\rho_{1}$(kg m^−3^). Symbols are experimental points, and lines are MT model fit.

**Figure 7:**
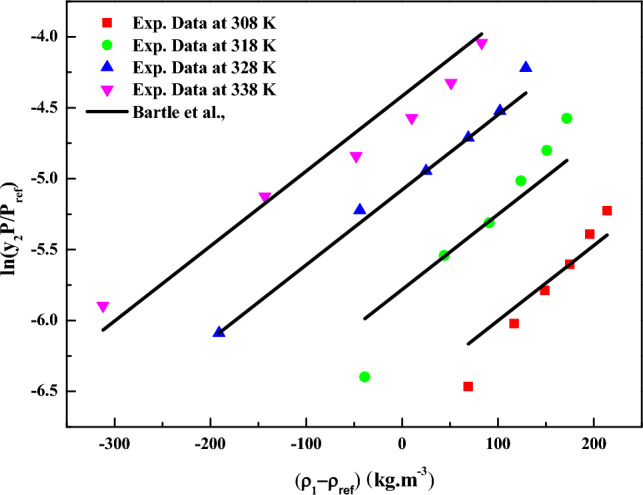
$\ln (y_{2} P/P_{ref} )$ versus $(\rho_{1} - \rho_{ref} )$ kg m^−3^. Symbols are experimental points, and lines are Bartle et al., model fit.

The results for data fitting of the *MT model* (Eq. 19) are presented in Fig. 6, and the corresponding parameters are reported in Table 4. The correlating ability of the *MT model* is evident in Fig. 5, where linear plots are observed when the data is plotted as ${T \mathord{\left/ {\vphantom {T {K\ln \left( {y_{2} \cdot P} \right)}}} \right. \kern-0pt} {K\ln \left( {y_{2} \cdot P} \right)}} - C_{2} T$ versus $\rho_{1}$ (Fig. 6), this further confirms the suitability of the *MT model* for the solubility data^72,73^. Similarly, the model proposed by *Bartle et al.* (Eq. 20) was correlated with solubility data, and the obtained results are reported in Table 4. Linear plots are also observed when the data is represented as $\ln \left( {{{y_{2} P} \mathord{\left/ {\vphantom {{y_{2} P} {P_{ref} }}} \right. \kern-0pt} {P_{ref} }}} \right)$ versus $\left( {\rho_{1} - \rho_{ref} } \right)$ (Fig. 7),confirming the applicability of the *Bartle *et al*.* model to the solubility data^72,73^.From the parameters of the *Bartle *et al*.* model, the vaporization enthalpy was determined, and its value is reported in Table 5.

**Figure 8:**
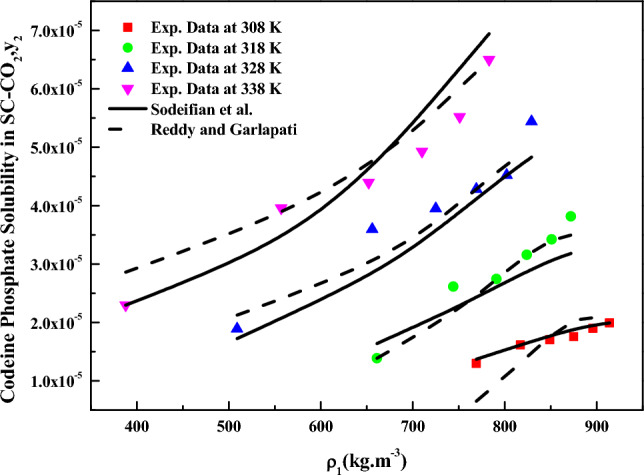
Codeine phosphate solubility in SC-CO_2_, $y_{2}$ versus $\rho_{1}$(kg m^−3^). Symbols are experimental points, lines are Sodeifian and Reddy-Garlapati model’s fit.

The solvation enthalpy was computed using the values of total and vaporization enthalpies, and the computed solvation enthalpy values are reported in Table 5. Notably, there is good agreement between the calculated average sublimation enthalpies from the *PR EoS* model (59.78 kJ/mol, as derived from Tables 3 and 5) and the calculated sublimation enthalpies from the *Bartle et al.* model (60.91 kJ/mol, as derived from Table 5). This suggests that using the PR EoS method in this study can yield meaningful correlation constants. However, the PR EoS accuracy decreases as the temperature increases from 308 to 338 K, possibly due to the temperature dependency of adjustable parameters. Figure 8 depicts the data fitting achieved using the *Sodeifian* and *Reddy–Garlapati* models.

**Figure 9:**
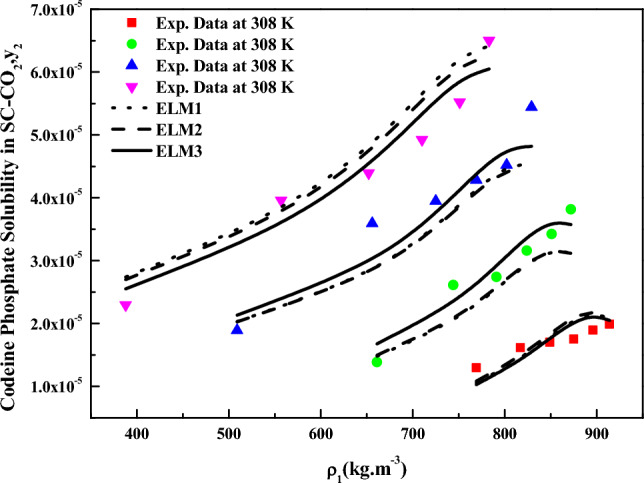
Codeine phosphate solubilities in SC-CO_2_, $y_{2}$ versus $\rho_{1}$(kg m^−3^). Symbols are experimental points; lines are ELMs model’s fit.

Three forms of expanded liquid models, precisely Eqs. (26), and (30), underwent evaluation with experimental data using the objective function mentioned in Eq. (12). Among these models, Eq. (30), which possesses the highest number of parameters, strongly agrees with the experimental data. Table 6 presents all the parameters associated with the expanded liquid models, and Fig. 9 shows the data correlation capabilities of these models.

**Table 6:** Correlation constant of ELM models.

Name of the model	Parameters	AARD%	$R^{2}$	$R_{adj}^{2}$
ELM1	$l_{1}$ = 10.134	11.1	0.919	0.890
	$l_{2}$ = − 598.37			
	$l_{3}$ = 31,626			
ELM2	$l_{1}$ = 10.196	11.0	0.919	0.891
	$l_{2}$ = − 600.81			
	$l_{3}$ = 31,962			
	$D$ = − 11.85			
ELM3	$l_{1}$ = 7.4325	8.89	0.952	0.935
	$l_{2}$ = − 609.58			
	$l_{3}$ = 32,270			
	$\beta_{1}$ = 224,920			
	$\beta_{2}$ = − 1269.3			
	$\beta_{3}$ = 13.166

The quality of data fit is contingent upon the number of parameters employed in the model. The Akaike Information Criterion (AIC) and the corrected AIC (AICc) are utilized to discern the optimal model. AIC_c_is computed based on *AIC*^74–77^, mathematical criteria commonly employed for assessing the compatibility of a solubility model with the corresponding solubility data. In statistics, these criteria compare solubility models and determine whichbest fits the data. *AIC* is appropriate when the data set comprises more than 40 data points, whereas *AIC*_*c*_ is preferred when the data set contains fewer than 40 data points^75,76^. The following is relation between AIC and AIC_c_. Additionally, the adjustable or mode parameters may be determined by different algorithms or methods such as nonlinear regression models^78,79^.

32 $$AIC_{c} = AIC + \frac{{2Q\left( {Q + 1} \right)}}{N - Q - 1}$$

In Eq. (32), *N* represents the number of experimental data points, *Q* denotes the adjustable constants of the model, and *AIC* is defined as the sum of $N\;\ln \left( {{{SSE} \mathord{\left/ {\vphantom {{SSE} N}} \right. \kern-0pt} N}} \right)$&$2Q$, where SSE stands for the sum of squared error. Table 7 displays all the computed values, revealing that Eq. (30) exhibits the lowest *AIC*_*c*_ value, establishing it as the most suitable model for the given data.

**Table 7:** Statistical values (AIC and AIC_c_) of all models.

Name of the model	RMSE	SSE	AIC	AIC_c_
EoS model	3.945 × 10^–6^	4.359 × 10^–10^	− 578	− 567.97
Chrastil	4.0383 × 10^–3^	3.5878 × 10^–4^	− 261	− 259.46
MT model	5.6176 × 10^–6^	6.9426 × 10^–10^	− 576	− 575.19
Bartle et al.	5.4034 × 10^–6^	6.4233 × 10^–10^	− 578	− 577.06
Sodeifian et al.	4.0748 × 10^–6^	3.6529 × 10^–10^	− 586	− 580.86
Reddy-Garlapati	3.3912 × 10^–6^	2.5301 × 10^–10^	− 595	− 587.67
ELM1	4.2894 × 10^–6^	4.4157 × 10^–10^	− 587	− 586.05
ELM2	4.2145 × 10^–6^	4.263 × 10^–10^	− 586	− 583.99
ELM3	3.2394 × 10^–6^	2.5185 × 10^–10^	− 595	− 589.79

The best model has the lowest AIC_c_ value. The six-parameter ELM model is identified as the optimal choice, while based on AIC_c_, the Chrastil model exhibits a weaker correlation than the other models considered in this study.

## Conclusion

This research presents, for the first time, solubility data of *codeine phosphate* in *SC-CO*_*2*_ measured at various conditions ranging between 308 and 338 K and 12–27 MPa. The measured data was found to vary within the range of (1.297–6.502) × 10^–5^ in mole fraction. The obtained solubility data were modeled using the *PREoS* model, with solute properties as one of the adjustable constants. Among the density models, the *Chrastil*, *MT*, and *Bartle et al.* models effectively captured the data. From the model constants, the enthalpies of the *SC-CO*_*2*_*-codeine phosphate* mixture were determined. Three expanded liquid models (*ELMs*) were applied to the solubility data. The model results indicate that all the *expanded liquid models (ELMs)* reasonably fit the data compared to the *PR EOS* and density models. Finally, AICc analysis indicates that the six-parameter *ELM model* is the most suitable model for data correlation. Considering the order of magnitude of solubility of *codeine phosphate* in *SC-CO*_*2*_, supercritical anti-solvent methods can be considered an appropriate choice for producing fine particles of this drug.

## Data Availability

Upon request, the data can be obtained from the corresponding author.

## List of symbols

$A_{{1}} ,B_{{1}}$: Chrastil’s model parameters (dimensionless, K)
$A_{{2}} ,B_{{2}} ,C_{{2}}$: MT model parameters (K, K m^3^/kg, dimensionless)
$A_{{3}} ,B_{{3}} ,C_{{3}}$: Bartle’s model parameters (dimensionless, K m^3^/kg)
$A_{{4}} ,B_{{4}} ,C_{{4}} ,D_{{4}} ,E_{{4}} ,F_{{5}}$: Sodeifian’s model parameters (dimensionless, K/MPa^2^, m^3^/kg K, m^6^/kg^2^, 1/K MPa, K m^3^/kg)
$A_{{5}} ,B_{{5}} ,C_{{5}} ,D_{{5}} ,E_{{5}} ,F_{{5}}$: Reddy-Garlapati’s model parameters (all are dimensionless)
AARD%: Average absolute relative deviation percentage
AIC: Akaike information criterion
AIC_c_: Corrected *AIC*
C_s_: The drug in the sample in vial (g/L)
D: Equation (27) parameter (J/molK)
E1 to E14: Symbols used in the experimental setup
${\text{H}}_{{{\text{sol}}}} ,{\text{H}}_{{{\text{sub}}}} ,{\text{H}}_{{{\text{total}}}}$: Enthalpy (J/mol or kJ/mol)
$K_{1} ,K_{2} ,K_{3}$: Equation (10) parameters (m^3^/mol, m^6^/mol kg, m^9^/mol kg^2^)
$l_{1} ,l_{2} ,l_{3}$: Activity coefficient parameters (dimensionless, J/mole MPa, J^2^/mole^2^ MPa^2^)
${\text{M}}_{{{\text{CO}}_{2} }} ,{\text{M}}_{{{\text{Solute}}}}$: The molar mass of CO_2_ and drug solute (g/mol)
$$n_{{{\text{CO}}_{2} }}$$: CO_2_ moles
$${n}_{d\mathrm{rug}}$$: Drug moles
*N*: Data points
NIST: National Institute of Standards and Technology
OF: Objective function
*Q*: Equation (32) parameter
P: Pressure (MPa)
${\text{P}}_{{\text{c}}} ,{\text{P}}_{{\text{r}}}$: Critical (Pa or MPa) and reduced pressures
Ps: Sublimation pressure (Pa or MPa)
*R*: Universal gas constant, 8.314 J/(mol K)
RMSD: Root mean square deviation
S: Solubility (kg/m_3_; kg mol/m_3_)
SSE: The sum of squares error
SC-CO_2_: Supercritical carbon dioxide
${\text{T}},{\text{T}}_{{\text{c}}}$: System temperature and critical temperature (K)
${v}_{1}{,v}_{2},{v}_{s}$: Molar volume (m^3^/mol)
$V_{{1}} ,V_{{\text{s}}}$: Sampling loop and collection vial volumes, in (μL)
${y}_{2}$: Drug solute solubility in mole fraction

## Greek symbols

$\beta$: Equation (11) parameters (dimensionless)
$\beta_{1} ,\beta_{2} \;{\text{and}}\;\beta_{3}$: Equation (30) parameters (J/K kg, J/K^2^kg and J/K^3^kg)
$$\gamma$$: Equation (11) parameters (K)
$\rho ,\rho_{r}$: Density (kg/m^3^, kgmol/m^3^), reduced density
$$\kappa$$: Association numbers
$\Delta_{sub} \delta$: Equation (11) parameters (dimensionless)

## Subscript

C: Critical
r: Reduced
Sol: Solvation
Sub: Sublimation
Total: Total

## Superscript

S: Saturation
